# First-Order Phase Transformation at Constant Volume: A Continuous Transition?

**DOI:** 10.3390/e24010031

**Published:** 2021-12-24

**Authors:** Víctor F. Correa, Facundo J. Castro

**Affiliations:** Consejo Nacional de Investigaciones Científicas y Técnicas (CONICET), Centro Atómico Bariloche (CNEA) and Instituto Balseiro (U. N. Cuyo), Av. Bustillo 9500, Bariloche 8400, Rio Negro, Argentina

**Keywords:** thermodynamics, first-order phase transition, isochoric process, water, mechanical actuator

## Abstract

We describe a first-order phase transition of a simple system in a process where the volume is kept constant. We show that, unlike what happens when the pressure is constant, (i) the transformation extends over a finite temperature (and pressure) range, (ii) each and every extensive potential (internal energy *U*, enthalpy *H*, Helmholtz energy *F*, and Gibbs energy *G*), and the entropy *S* is continuous across the transition, and (iii) the constant-volume heat capacity does not diverge during the transition and only exhibits discrete jumps. These non-intuitive results highlight the importance of controlling the correct variables in order to distinguish between continuous and discontinuous transitions. We apply our results to describe the transition between ice VI and liquid water using thermodynamic information available in the literature and also to show that a first-order phase transition driven in isochoric condition can be used as the operating principle of a mechanical actuator.

## 1. Introduction

Phase transitions (PT) are probably one of the most interesting and conceptually rich phenomena approached by Thermodynamics and Statistical Mechanics. The classical traditional classification presented in 1933 by P. Ehrenfest [1] introduces the concept of transition order: When at least one of the first order derivatives of the Gibbs energy G(T,p) with respect to its natural variables, temperature *T*, and pressure *p*, shows a jump discontinuity, the transition is said to be first order; if all the first-order derivatives are continuous but at least one of the second-order derivatives shows a jump discontinuity, the transition is said to be second order, and so on for higher-than-second-order transitions. Since then, this scheme has become universally accepted due to its simplicity and conceptual content. Even after the development of modern critical phenomena theories and related concepts such as order parameter, correlation length, fluctuations, and symmetry, the classification has remained valid in a simplified form: first-order or discontinuous transitions on one side and continuous transitions on the other [2]. Though the classification was originally thought for a simple system characterized by the variables *T*, *p*, and the volume *V* (of which only two are independent), it can be generalized to include other variables such as electric and magnetic fields or strain–stress effects, as long as the system remains thermodynamic: large enough as to neglect surface and geometrical effects and without long-range interactions that could invalidate the additive nature of the extensive variables.

From the experimental point of view, the first studies on PT conducted at constant pressure and temperature realized the presence of a latent heat *L* and/or a volume change ΔV across the transition. In fact, it was the lack of any observable *L* or ΔV in the, at that time, newly discovered superfluid transition what triggered the Ehrenfest work [2]. As the latent heat *L* can be connected with the entropy *S* ($L=T_{0}\Delta S$, where $T_{0}$ is the transition temperature), and entropy and volume *V* are first-order derivatives of the Gibbs energy, it seems natural to give a classification in terms of G(T,p).

In addition to this, there are additional reasons that point towards G(T,p) as a special thermodynamic potential to analyze PT. These reasons naturally arise when examining phase transitions under different experimental conditions. A first scenario to do this is constant volume and temperature, but we will show in the following that a textbook first-order transition, with finite *L* and ΔV, in a simple system cannot take place under constant *V* and *T* conditions. A second scenario is constant volume only. We will examine this process in detail and demonstrate that: (i) the transformation extends over a finite range of *T* (and *p*), and (ii) each and every extensive potential (internal energy *U*, enthalpy *H*, Helmholtz energy *F*, and Gibbs energy *G*) and the entropy *S* are continuous across the transition when *V* is constant. After carefully analyzing the details of this transition two examples will be presented: (1) a possible constant volume transformation between liquid water and ice VI and (2) a process analogous to a mechanocaloric cycle but based on a constant volume phase transformation that could be used as a mechanical actuator that does work on a external system by changing the temperature.

## 2. Recapitulation of a Constant Pressure Transformation

**Figure 1:**
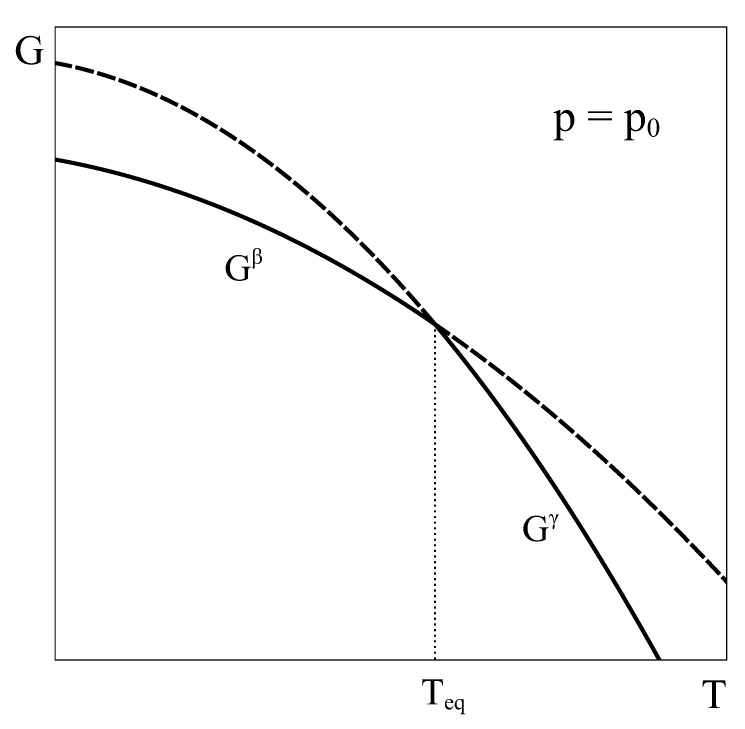
Qualitative Gibbs energy curves of phases β and γ as a function of temperature at a constant pressure $p_{0}$. Both phases coexist in equilibrium at $T=T_{eq}$. Solid (dash) lines correspond to stable (metastable) states.

Let us begin by recalling phase equilibrium and the characteristics of a first-order transition at constant $p=p_{0}$. Consider a simple system with a well-defined composition and completely characterized by the variables *p*, *T*, and V(T,p). This macroscopic system can exist in two different phases (β and γ) in the domain of interest of the p−T phase diagram, and the transformation between these phases is a first-order transformation, with finite enthalpy and volume changes associated with it. The classical analysis of phase equilibrium in this case is typically made by plotting the characteristic Gibbs functions of phases β and γ as a function of temperature at the constant pressure $p_{0}$, as shown in Figure 1.

At each temperature *T*, the state of equilibrium of the system corresponds to the phase that has the lower Gibbs energy, as equilibrium under constant *T* and *p* corresponds to the state that minimizes the Gibbs energy. Therefore, for temperatures below $T_{eq}$, β is the stable phase, and above $T_{eq}$, γ is the stable phase. At $T=T_{eq}$, the Gibbs energy curves coincide, and phases β and γ can coexist in equilibrium (Figure 1). Hence, if the system is slowly heated at constant pressure $p_{0}$ starting in an equilibrium state in the β single phase field at a temperature $T_{0}$, it keeps in this phase until temperature $T_{eq}$ is reached. At this temperature, phase transformation under equilibrium begins, and the system gradually moves from the β phase to the γ phase. During this transformation, heat evolves according to the enthalpy difference between phases β and γ, and the volume of the system changes following the differences of molar volumes. The finite change of slope in passing from the Gibbs energy of β phase to that of γ corresponds to the finite entropy change characteristic of this first-order phase transformation.

To further analyze the transition, it is useful to employ the concept of extent of reaction (ξ). This parameter, first introduced by T. De Donder in 1920, is very helpful to describe the amount of each reactive and product by a single parameter in a chemical reaction taking place within a closed vessel [3]. The extent of the reaction can also be used to analyze the evolution of a phase transition by considering the transformation from β phase to γ phase as the following physicochemical reaction:

(1) β⇌γ.

By means of ξ, the number of moles of each phase is given by:

(2) $$n^{\beta }=1-\xi \;\;n^{\gamma }=\xi ,$$

where, for simplicity, we are considering 1 mole of substance. Every extensive quantity of the complete system can be expressed in terms of ξ and the corresponding molar quantity. For example, the system volume and enthalpy are given by:

(3) $$\begin{array}{ccc} V(T,p) & = & V_{m}^{\beta }\left(T,p\right)\;n^{\beta }+V_{m}^{\gamma }\left(T,p\right)\;n^{\gamma }\\ & = & V_{m}^{\beta }\left(T,p\right)+\left[V_{m}^{\gamma }\left(T,p\right)-V_{m}^{\beta }\left(T,p\right)\right]\;\xi ,\\ & = & V_{m}^{\beta }\left(T,p\right)+\Delta V_{m}^{PT}\left(T,p\right)\xi , \end{array}$$

(4) $$\begin{array}{ccc} H(T,p) & = & H_{m}^{\beta }\left(T,p\right)\;n^{\beta }+H_{m}^{\gamma }\left(T,p\right)\;n^{\gamma }\\ & = & H_{m}^{\beta }\left(T,p\right)+\left[H_{m}^{\gamma }\left(T,p\right)-H_{m}^{\beta }\left(T,p\right)\right]\;\xi ,\\ & = & H_{m}^{\beta }\left(T,p\right)+\Delta H_{m}^{PT}\left(T,p\right)\xi . \end{array}$$

where $\Delta V_{m}^{PT}\left(T,p\right)$ and $\Delta H_{m}^{PT}\left(T,p\right)=L$ denote the volume and enthalpy changes (latent heat) associated with the phase transformation, respectively. In fact, by measuring the heat taken or released by the system (or the volume) during the phase transformation, the value of ξ can be inferred, and from it, the value of any extensive quantity of the system can be calculated by using expressions similar to Equations (3) and (4).

If the enthalpy of the complete system is analyzed as a function of temperature, at $T=T_{eq}$, the characteristic jump associated with the latent heat is found. It is interesting to note that the jump or discontinuity is associated with the complete phase transformation. During the transformation, continuous values of enthalpy can be attributed to the system at $T=T_{eq}$ by means of the extent of transformation ξ following Equation (4). The temperature derivative of this curve corresponds to the constant pressure heat capacity of the system (defined by $C_{p}={\left(\frac{\partial H}{\partial T}\right)}_{p}=T{\left(\frac{\partial S}{\partial T}\right)}_{p}$). The limits of this expression reaching the transition point from the left or from the right are different because they correspond to the temperature derivative of the enthalpy of β or γ phase, respectively. Additionally, during the transition, there is a finite enthalpy change with no associated temperature change. Therefore, there is a divergence in $C_{P}$ that, in an ideal representation of an equilibrium phase transformation, can be described by a Dirac δ-function. A similar reasoning shows that the volume thermal-expansion coefficient $\alpha =\frac{1}{V}{\left(\frac{\partial V}{\partial T}\right)}_{p}$ and the isothermal compressibility $\kappa =-\frac{1}{V}{\left(\frac{\partial V}{\partial p}\right)}_{T}$ exhibit similar behavior during a constant pressure transition.

## 3. Constant Volume Transformation

### 3.1. General Properties

**Figure 2:**
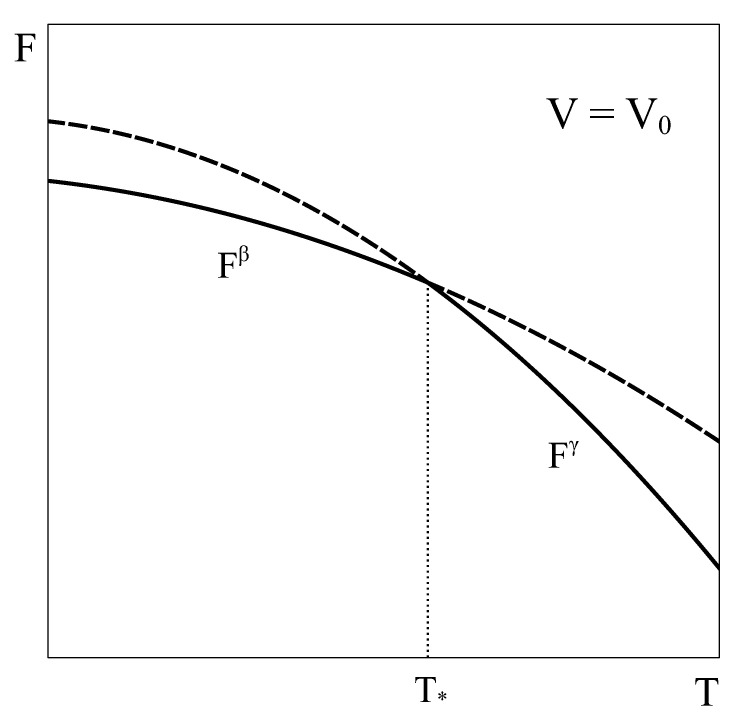
Schematic Helmholtz energy curves of phases β and γ as a function of temperature at a constant volume $V_{0}$. At $T=T_{*}$, the energies of both phases match which, in general, is not associated with a phase transition. See text for details.

Let us now consider an approach similar to the previous one, but applied to a constant volume transition. As the equilibrium of the system under constant *T* and *V* is given by the minimum of the Helmholtz potential, let us schematically represent typical Helmholtz curves of phases β and γ as a function of temperature under constant $V=V_{0}$ conditions (Figure 2).

By following a similar reasoning to that of Section 2, it could be concluded that phase β is the stable phase below temperature $T_{*}$ and phase γ is the stable one above it. Additionally, it could be thought that at $T_{*}$, both phases could coexist in equilibrium. However, this argument has two flaws. First, nothing ensures that at volume $V_{0}$ and $T=T_{*}$ the pressure of phase β given by its equation of state matches that of phase γ. In fact, in general, this condition is not met. Therefore, the equality of all the characteristic intensive variables of each phase, a necessary requirement for the equilibrium of two phases, would not be satisfied [4,5]. Secondly, nothing ensures that there is no other Helmholtz energy curve that fulfills the constant volume condition and also lies below both single-phase Helmholtz energy curves, so giving the actual minimum. This could be achieved, for example, by combining different amounts of phases β and γ at each temperature, thanks to the fact that volume is an extensive quantity. In fact, in the following we will see that such a curve indeed exists!

This analogy highlights other aspects of the key role played by the Gibbs energy. First, by exclusively depending on the intensive variables *p* and *T*, it can be assured that when two Gibbs energy curves at constant p intersect at an equilibrium temperature (or two curves at constant *T* intersect at an equilibrium pressure) the other intensive variable also coincides, ensuring equilibrium. Secondly, the absence of natural extensive variables of *G* precludes the possibility of combining two single phase Gibbs energy curves into a new curve that could lie below both single-phase characteristic G curves.

**Figure 3:**
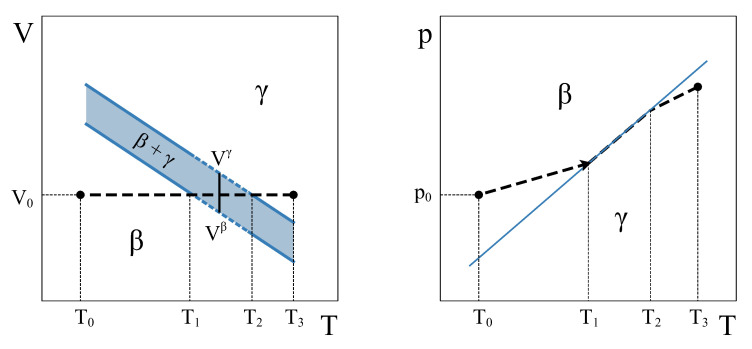
Volume–Temperature (**left**) and Pressure–Temperature (**right**) schematic phase diagrams of a simple system. Two phases, β and γ, transform each other via a first-order transition. The dash-line corresponds to a constant volume process starting at $V_{0}\left(T_{0},p_{0}\right)$.

Therefore, how can a constant volume phase transformation be analyzed? The first thing to notice is that a constant volume first-order transformation must occur over a temperature range. To see this, let us go back to our simple system analyzed in Section 2. A V−T phase diagram for this system (Figure 3, left panel) shows two single-phase regions and an area in the V−T plane where β and γ phases coexist. The existence of an area instead of a line is a consequence of the finite jump in volume at each temperature associated with the first order PT. If we want the system now to transform from phase β to phase γ at a constant volume $V_{0}$, the temperature during the phase transformation must necessarily change. By starting in the β single-phase field at $V_{0}\left(T_{0},p_{0}\right)$ and warming up the system at constant volume, γ will start nucleating at $T_{1}$, but the phase transformation will not end until temperature $T_{2}$ is reached (Figure 3). During the coexistence, both phases must be at equilibrium. This condition requires the equality of temperature, pressure, and chemical potential [4,5]. These conditions are met if the system evolves along the p−T coexistence line in the p−T phase diagram (Figure 3, right panel). Therefore, during phase coexistence at constant volume, *p* and *T* change and are linked by the coexistence line. As the volume is fixed by the isochoric condition, only one degree of freedom survives.

Algebraically, the need for phase coexistence to occur over a finite range of temperatures can be seen by taking the differential of the system volume (3) and imposing the constant volume condition:

(5) $$0=dV=dV_{m}^{\beta }\left(T,p\right)\;n^{\beta }+dV_{m}^{\gamma }\left(T,p\right)\;n^{\gamma }+V_{m}^{\beta }\left(T,p\right)\;dn^{\beta }+V_{m}^{\gamma }\left(T,p\right)\;dn^{\gamma }.$$

Using the extent of transformation ξ (Equation (2)), the volume thermal-expansion coefficient α and the isothermal compressibility κ for each phase, Equation (5) can be rewritten as:

(6) $$0=\left(\alpha^{\beta }V_{m}^{\beta }n^{\beta }+\alpha^{\gamma }V_{m}^{\gamma }n^{\gamma }\right)dT-\left(\kappa^{\beta }V_{m}^{\beta }n^{\beta }+\kappa^{\gamma }V_{m}^{\gamma }n^{\gamma }\right)dp+\left(V_{m}^{\gamma }-V_{m}^{\beta }\right)d\xi ,$$

where, for readability, the dependence of α, κ, and the molar volumes on pressure and temperature has not been explicitly written. As we mentioned before, the equilibrium between the β and γ phases during the transition makes temperature and pressure non-independent quantities. At each temperature, the pressure is given by the coexistence line $p=p_{c}\left(T\right)$. Therefore, Equation (6) can be rewritten as:

(7) $$\begin{array}{ccc} 0 & = & \left[\alpha^{\beta }V_{m}^{\beta }n^{\beta }+\alpha^{\gamma }V_{m}^{\gamma }n^{\gamma }-\left(\kappa^{\beta }V_{m}^{\beta }n^{\beta }+\kappa^{\gamma }V_{m}^{\gamma }n^{\gamma }\right)\frac{dp_{c}}{dT}\right]dT\\ & & +(V_{m}^{\gamma }-V_{m}^{\beta })d\xi . \end{array}$$

We can see here that for the constant volume transformation to advance (dξ≠0), the temperature (and pressure) must change (dT≠0). In other words, as the β and γ molar volumes are different, due to the first-order nature of the transition, the only way of keeping the volume constant during a phase transformation is by changing temperature and pressure in order to compensate for the differences in molar volumes. Therefore, in a general isochoric first-order transformation, the transition takes place over a temperature range. Only in the very special case of two phases with no volume change across the transition, i.e., if $V_{m}^{\beta }\left(T_{*}\right)$ = $V_{m}^{\gamma }\left(T_{*}\right)$ = $V_{0}$, the transformation would take place at a single equilibrium temperature $T_{*}$ (as sketched in Figure 2). As mentioned before, in a general isochoric transformation, the temperature and pressure coexistence range poses no problem to the equilibrium conditions. The evolution along the coexistence p−T curve ensures the equality of the chemical potentials of β and γ.

During the transition, the extent of reaction ξ can be expressed as a function of *T*. By imposing the constant volume condition in Equation (3) and taking into account that during the transition $p=p_{c}\left(T\right)$, ξ can be expressed as:

(8) $$\xi \left(T\right)=\frac{V_{0}-V_{m}^{\beta }\left[T,p_{c}\left(T\right)\right]}{V_{m}^{\gamma }\left[T,p_{c}\left(T\right)\right]-V_{m}^{\beta }\left[T,p_{c}\left(T\right)\right]}=\frac{V_{0}-V_{m}^{\beta }\left(T\right)}{\Delta V_{m}^{PT}\left(T\right)}.$$

Two straightforward conclusions can be drawn from this equation: (i) $\xi (T_{1})=$ 0 and $\xi (T_{2})=$ 1, since $V_{m}^{\beta }\left(T_{1}\right)=V_{0}$ and $V_{m}^{\gamma }\left(T_{2}\right)=V_{0}$, respectively; (ii) ξ(T) is a continuous function since $V_{0}$ is constant and the molar volumes of both phases are continuous functions.

Between $T_{1}$ and $T_{2}$, the mole numbers of each phase are explicitly given by

(9) $$n^{\beta }\left(T\right)=\frac{V_{m}^{\gamma }\left(T\right)-V}{V_{m}^{\gamma }\left(T\right)-V_{m}^{\beta }\left(T\right)}\;\;n^{\gamma }\left(T\right)=\frac{V-V_{m}^{\beta }\left(T\right)}{V_{m}^{\gamma }\left(T\right)-V_{m}^{\beta }\left(T\right)}.$$

### 3.2. Calculation of Thermodynamic Quantities

Let us consider now a general extensive variable *Z* and calculate its value during the coexistence at constant $V=V_{0}$. According to Euler’s theorem for homogeneous functions [4], *Z* can be calculated as:

(10) $$Z\left(T\right)=Z\left[p_{c}\left(T\right),T\right]=n^{\beta }\;Z_{m}^{\beta }\left[p_{c}\left(T\right),T\right]+n^{\gamma }\;Z_{m}^{\gamma }\left[p_{c}\left(T\right),T\right],$$

which in terms of the extent of transformation reads:

(11) $$\begin{array}{ccc} Z(T) & = & Z_{m}^{\beta }\left(T\right)+\left[Z_{m}^{\gamma }\left(T\right)-Z_{m}^{\beta }\left(T\right)\right]\xi \left(T\right)\\ & = & Z_{m}^{\beta }\left(T\right)+\Delta_{PT}Z_{m}\left(T\right)\;\xi \left(T\right). \end{array}$$

Here, $\Delta_{PT}Z_{m}\left(T\right)=Z_{m}^{\gamma }\left(T\right)-Z_{m}^{\beta }\left(T\right)$ represents the change of $Z_{m}$ across the transformation. If we take $Z\left(T_{1}\right)=Z_{m}^{\beta }\left(T_{1}\right)$ as a reference state and calculate the change in Z from this reference state, we obtain:

(12) $$\Delta Z\left(T\right)=Z\left(T\right)-Z_{m}^{\beta }\left(T_{1}\right)=Z_{m}^{\beta }\left(T\right)-Z_{m}^{\beta }\left(T_{1}\right)+\Delta_{PT}Z_{m}\left(T\right)\;\xi \left(T\right).$$

We can identify two contributions to ΔZ(T): the change of $Z_{m}^{\beta }$ due to the variation of *T* and the change due to the phase transformation itself. A quick overview of this equation allows us to see that ΔZ(T) is continuous as long as $Z_{m}^{i}$ (i=β,γ) is well defined and continuous in the whole temperature range, and this is indeed true for each and every extensive potential: U,F,H,G, and the entropy *S*. In other words, during a phase transformation at constant volume, there are no jumps or discontinuities in any thermodynamic potential at any single temperature. This might seem rather puzzling at a first glance, as the transformation appears as a continuous one. However, this appearance is nothing but the consequence of the transformation to extend over a finite temperature (and pressure) range. The characteristic jump of the first-order transition is spread over a temperature range when the transformation is conducted at constant volume, making it appear as if it were a continuous change.

As examples, Equation (12) can be applied to the cases of the Helmholtz potential *F* and the entropy *S*. For F(T,V), the contribution from the transformation is:

(13) $$\Delta_{PT}F_{m}\left(T\right)=\Delta_{PT}\left[G_{m}\left(T\right)-p_{c}\left(T\right)V_{m}\left(T\right)\right]=-p_{c}\left(T\right)\Delta_{PT}V_{m}\left(T\right),$$

where we have used the equality of the chemical potentials during the transformation $G_{m}^{\beta }=G_{m}^{\gamma }$. By adding the first two terms of Equation (12), we finally obtain:

(14) $$\Delta F\left(T\right)=F_{m}^{\beta }\left(T\right)-F_{m}^{\beta }\left(T_{1}\right)-p_{c}\left(T\right)\Delta_{PT}V_{m}\left(T\right)\;\xi \left(T\right).$$

Proceeding with the entropy, from the Clapeyron equation, we obtain:

(15) $$\Delta_{PT}S_{m}\left(T\right)=\Delta_{PT}V_{m}\left(T\right)\frac{dp_{c}\left(T\right)}{dT},$$

so the entropy change is:

(16) $$\Delta S\left(T\right)={\left(\frac{\partial F_{m}^{\beta }}{\partial T}\right)}_{V_{m}^{\beta }=V}\left(T_{1}\right)-{\left(\frac{\partial F_{m}^{\beta }}{\partial T}\right)}_{V_{m}^{\beta }=V}\left(T\right)+\Delta_{PT}V_{m}\left(T\right)\frac{dp_{c}\left(T\right)}{dT}\;\xi \left(T\right).$$

It is easy to check that the same result is obtained by the differentiation of Equation (14), i.e., $\Delta S\left(T\right)={\left(\frac{\partial \Delta F}{\partial T}\right)}_{V}-S_{m}^{\beta }\left(T_{1}\right)$. Additionally, it is interesting to note that all the previous expressions can be explicitly calculated if the quantities $F_{m}^{i}\left(T,V\right)$, $V_{m}^{i}$, and $p_{c}\left(T\right)$ are known. In Appendix A, the expressions for *U*, *H*, and *G* in terms of the same quantities are given.

We have seen in Section 2 that the constant pressure heat capacity exhibits a divergence during a first-order transformation at constant pressure. The analysis previously conducted on a constant volume transformation allows us to examine what happens to the heat capacity at constant volume during the isochoric transition. Contrary to what happens in a constant pressure PT, in a constant volume PT we have shown that all the thermodynamic potentials and the entropy are continuous functions across the transformation. Therefore, no divergences are expected in this case. However, there can be discrete jumps in heat capacity if different curvatures are met during the transformation.

Following Equation (10), the heat capacity at constant volume of the whole system (β and γ phases) $C_{V}$ can be expressed as:

(17) $$C_{V}=n^{\beta }c_{V}^{\beta }+n^{\gamma }c_{V}^{\gamma },$$

where $c_{V}^{i}$ (i=β,γ) are the molar specific heats for each phase at constant total volume *V* ($c_{V}^{i}$ takes into account not only the internal energy change due to a change of temperature but also the one due to a variation of $n^{i}$ in the transformation (see Appendix B)). After some algebra (see Appendix B), we arrive at the following expression:

(18) $$C_{V}=n^{\beta }\left[c_{V_{m}^{\beta }}^{\beta }+T\;V_{m}^{\beta }\;\kappa^{\beta }{\left(\frac{\alpha^{\beta }}{\kappa^{\beta }}-\frac{dp_{c}}{dT}\right)}^{2}\right]+\;n^{\gamma }\left[c_{V_{m}^{\gamma }}^{\gamma }+T\;V_{m}^{\gamma }\;\kappa^{\gamma }{\left(\frac{\alpha^{\gamma }}{\kappa^{\gamma }}-\frac{dp_{c}}{dT}\right)}^{2}\right],$$

which is valid on the coexistence line. $c_{V_{m}^{i}}^{i}={\left(\frac{\partial U_{m}^{i}}{\partial T}\right)}_{V_{m}^{i}}$ (i=β,γ) denotes the molar specific heat at constant molar volume of the *i*-phase. We can evaluate this expression at the onset of the transformation, $T_{1}$, where $n\to n^{\beta }$ and $V\to V_{m}^{\beta }$. As $T\to T_{1}^{+}$, on the coexistence field, $\frac{dp_{c}}{dT}=\frac{\Delta_{PT}S_{m}}{\Delta_{PT}V_{m}}$. Thus:

(19) $$\lim_{T\to T_{i}^{+}}C_{V}=n^{\beta }\left[c_{V_{m}^{\beta }}^{\beta }+T\;V_{m}^{\beta }\;\kappa^{\beta }{\left(\frac{\alpha^{\beta }}{\kappa^{\beta }}-\frac{\Delta_{PT}S_{m}}{\Delta_{PT}V_{m}}\right)}^{2}\right].$$

On the other hand, as $T\to T_{1}^{-}$, on the single-phase β field, the limit is trivial:

(20) $$\lim_{T\to T_{1}^{-}}C_{V}=n^{\beta }c_{V_{m}^{\beta }}^{\beta }.$$

It is clear from Equations (19) and (20) that $C_{V}$ is discontinuous at $T_{1}$ (and at $T_{2}$, as a similar reasoning shows). Heat added to the system in the coexistence region not only is used to raise *T* but to phase-transform as well. This is the origin of the discontinuity. During the constant *V* transformation, as the system traverses the p−T coexistence line, the other susceptibilities (α, κ, and $C_{p}$) are strictly not defined (the limits from either side do not match) or can be thought of as divergences due to the finite volume or enthalpy changes without changes in pressure or temperature. Summarizing, during the constant *V* transformation, finite jumps are observed in $C_{V}$ only when entering and leaving the coexistence region, whereas $C_{p}$, α, and κ are not defined within the coexistence region.

## 4. Application 1: The Liquid–Ice VI Transition in Water

**Figure 4:**
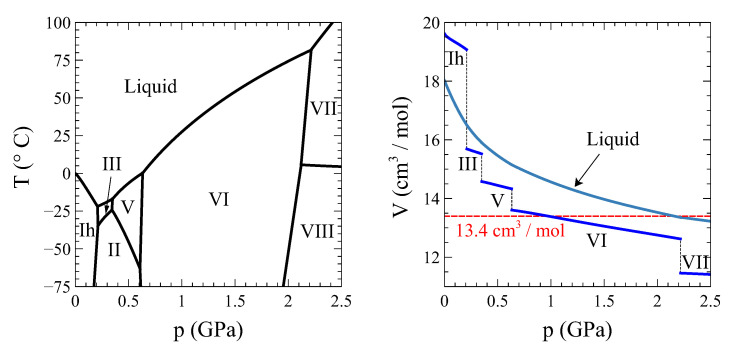
Temperature-pressure phase diagram of water (**left**) and the molar volume of liquid and solid water along the phase coexistence line (**right**). Data taken from [6].

A sine qua non condition for the possibility of a full transformation at constant volume is that the starting and final phases share the same molar volume. The bell-shaped coexistence region between vapor and liquid water in the p−V diagram precludes this possibility. Figure 4 shows the temperature vs. high-pressure phase diagram of water (left panel) together with a molar volume vs. pressure plot of the different ice phases and liquid water along the coexistence lines (right panel). It is clear from this figure that the only transition that meets the requirement of equal-initial-final volume and involves two phases only (i.e., no transitions to intermediate phases are required, as it will be the case for example with the ice V to liquid water transition) is the liquid–ice VI one.

**Figure 5:**
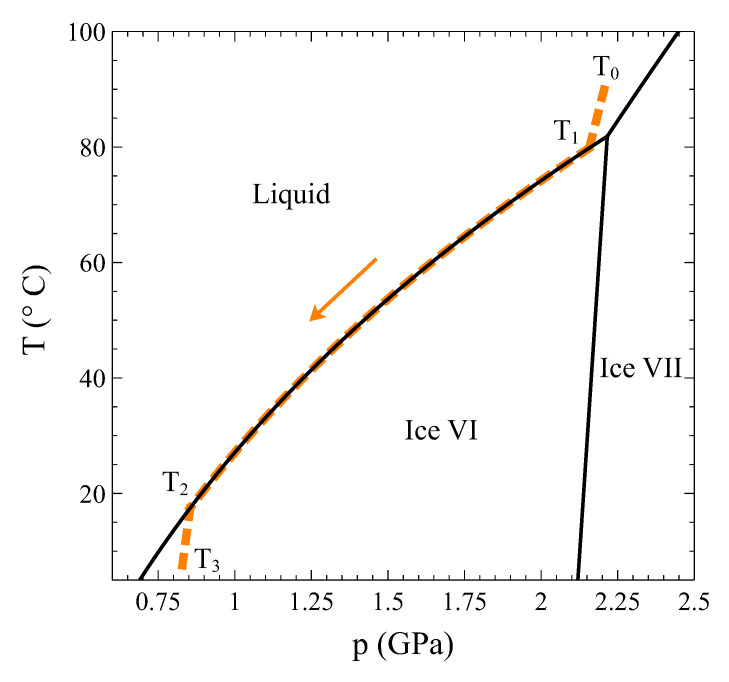
Temperature–pressure phase diagram of water around the liquid–ice VI transition. The dashed colored line is the trajectory of a process at constant volume $V_{0}=$ 13.4 cm${}^{3}$/mol starting in the liquid phase ($T_{0}$) and ending up in the ice phase ($T_{3}$). The two phases coexist between $T_{1}$ and $T_{2}$.

So, let us study this transition in detail. Using the complete thermodynamic information of liquid water [7], ice VI [8,9], and the coexistence line between both phases [10], we have calculated the trajectory of a process at constant volume $V_{0}=$ 13.4 cm${}^{3}$/mol that starts at $T_{0}$ = 90 °C in the liquid phase, is cooled through the transition, and ends up at $T_{3}$ = 7 °C in the ice VI phase (Figure 5). Particularly, at $T_{1}$ = 79.81 °C, the liquid phase begins to transform into ice VI, and finally, at $T_{2}$ = 17.45 °C, the transformation completes.

**Figure 6:**
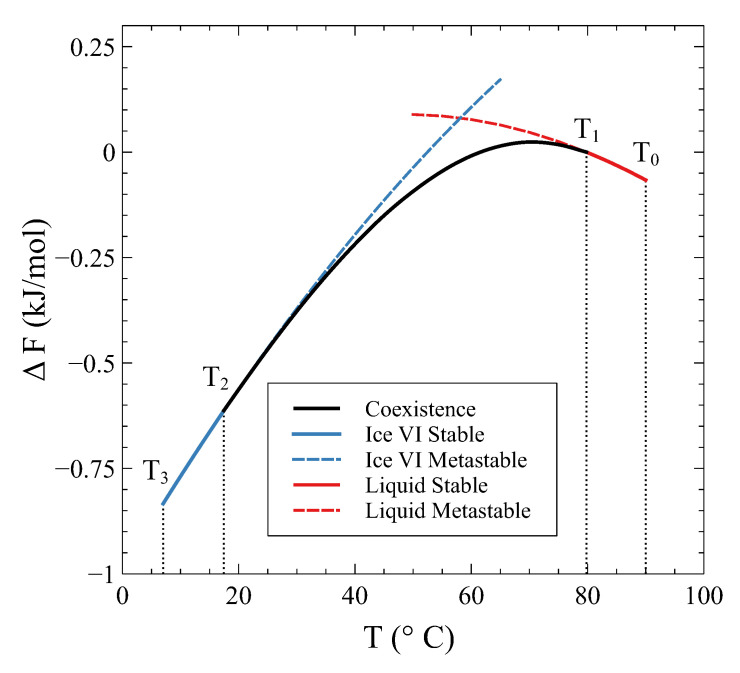
The Helmholtz energy change $\Delta F\left(T\right)=F\left(T\right)-F_{m}^{liq}\left(T_{1}\right)$ in a process at constant volume $V_{0}=$ 13.4 cm${}^{3}$/mol. The dashed lines are the metastable parts of the single-phase curves.

The Helmholtz energy change $\Delta F\left(T\right)=F\left(T\right)-F_{m}^{liq}\left(T_{1}\right)$ along this constant volume path is displayed in Figure 6. The dashed lines correspond to the extrapolation of the single-phase curves into the coexistence region.

First, it can be seen that *F* values continuously change during the constant *V* transformation. Secondly, it can be appreciated that the *F* curve associated with the coexistence of β and γ phases always lies below the single phase *F* curves. This is the combination of the single phase *F* curves previously mentioned in Section 3 that minimizes the Helmholtz energy at constant *V* and at each *T* in the coexistence region.

**Figure 7:**
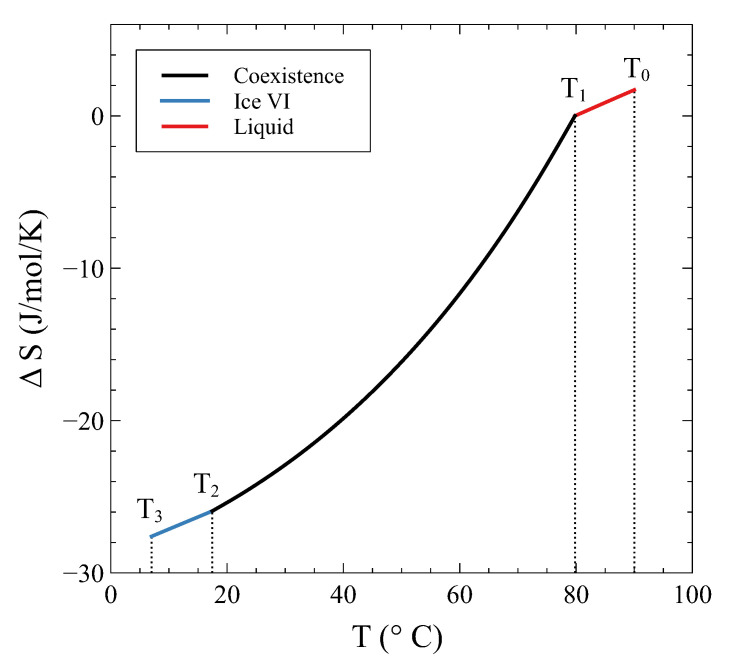
The entropy change $\Delta S\left(T\right)=S\left(T\right)-S_{m}^{liq}\left(T_{1}\right)$ in a process at constant volume $V_{0}=$ 13.4 cm${}^{3}$/mol.

Not only ΔF is continuous at $T_{1}$ and $T_{2}$, but its derivative is continuous, too. This derivative is nothing but (minus) the entropy, which is shown in Figure 7 as $\Delta S\left(T\right)=S\left(T\right)-S_{m}^{liq}\left(T_{1}\right)$.

**Figure 8:**
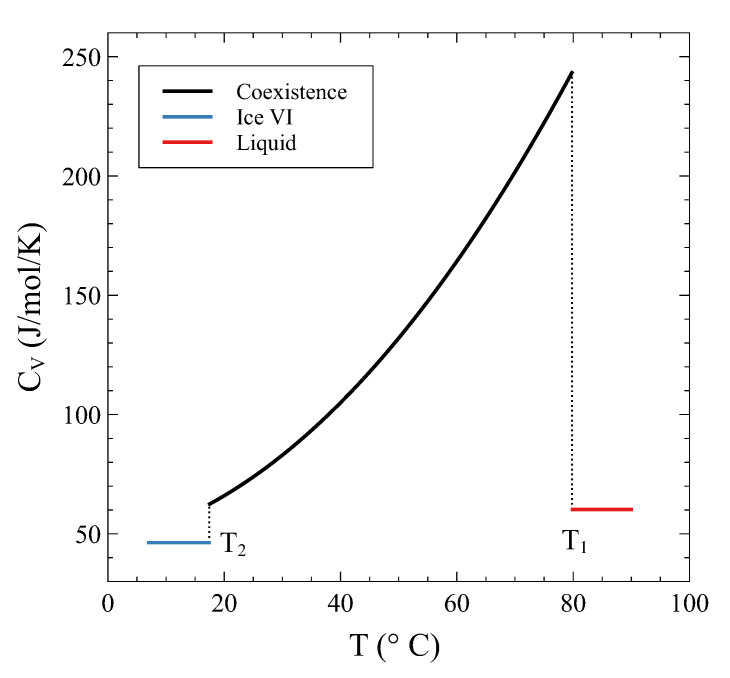
Heat capacity at constant volume $V_{0}=$ 13.4 cm${}^{3}$/mol as a function of temperature. Jumps can be seen when entering and leaving the coexistence region.

Its continuity during the transition has been previously mentioned and can be explicitly seen in this example. On the other hand, the derivative of the entropy, i.e., $C_{V}/T$, is clearly discontinuous at $T_{1}$ and $T_{2}$, as has been anticipated. Figure 8 shows $C_{V}$ across the transition.

The behavior of G,U, and *H* can be found as supplementary information.

## 5. Application 2: Temperature-Driven Mechanical Actuator

Similarly to the constant volume process described here, it is also possible to go through a first-order phase transition in a constant entropy process. This is the basis of the so-called caloric effects: the temperature change in an adiabatic system associated with the application of an external stress or pressure (mechanocaloric), an external magnetic field (magnetocaloric), or an external electric field (electrocaloric). The effects have been largely studied due to their potential applications in refrigeration technologies [11,12,13,14,15].

The whole idea is quite simple. For instance, in the mechanocaloric effect, a substance with a pressure-dependent entropy (the refrigerant) cools down when the applied pressure is adiabatically released. The refrigerant is then put in contact with the system to be refrigerated in a cyclic process. The higher performance, i.e., the largest temperature change for a given applied pressure, is obtained when the refrigerant goes through a first-order transition during the adiabatic stage and is a consequence of the associated latent heat. Giant mechanocaloric effects around room temperature have been reported for Cu-based and Ni–Ti-based families of martensitic alloys where, typically, temperature changes of some tens of degrees Celsius are obtained with applied pressures of some hundreds of MPa [11].

**Figure 9:**
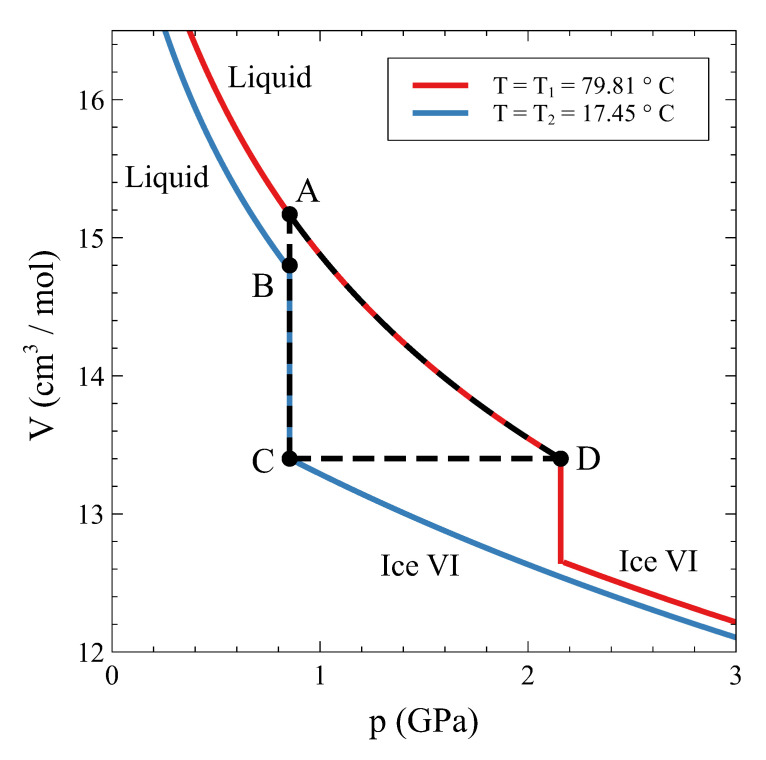
Cycle for a mechanical actuator using ordinary water as the working substance and the isochoric phase transition between liquid water and ice VI.

In a similar way, a substance that shows a first-order phase transformation can be used as a mechanical actuator in a cycle that involves an isochoric step. The working principle is sketched in Figure 9. Let us suppose that the operative substance is ordinary water driven through the liquid–ice VI transition. Starting in the liquid phase at $T_{1}=$ 79.81 °C and $p_{c}\left(T_{2}\right)$ (state A in Figure 9), water is isobarically cooled down to $T_{2}=$ 17.45 °C (state B). From this state, liquid water is made to fully transform isothermically and isobarically to ice VI (state C). After that, the substance is isochorically driven from $T_{2}$ to $T_{1}$ along the coexistence line, and when state D is reached, ice VI has fully transformed to liquid water again. Along the C–D step, the pressure goes up to $p_{c}\left(T_{1}\right)$, an approximate increment of 1.3 GPa (see Figure 9). This pressure change can be used to deliver work to an external system, a positioner, for instance. Eventually, the substance returns to state A, and the cycle may start over.

The performance of such a device is quite interesting. A temperature variation of about 60 °C results in a usable pressure change ($p_{c}\left(T_{1}\right)$ − $p_{c}\left(T_{2}\right)$) of about 1.3 GPa. In the best scenario, this implies that a volume change V(C)−V(A)∼ 1.8 cm${}^{3}$/mol is available for usage after each cycle. In a linear actuator, a maximum relative displacement $∼\frac{\Delta V}{V}∼$ 0.12 can be attained. While in the mechanocaloric effect, the external parameter controlling the refrigeration is pressure, in this actuator, the control parameter that delivers external work is temperature.

## 6. Conclusions

As conclusion, we want to remark that we have extensively described the thermodynamics of a constant volume phase transition for a simple system that exhibits a first-order phase transformation. With our approach, we have shown that (i) the transformation extends over a finite range of *T* (and *p*); (ii) each and every extensive potential (internal energy *U*, enthalpy *H*, Helmholtz energy *F*, and Gibbs energy *G*) and the entropy *S* are continuous across the transition, unlike what is observed in a constant pressure (and temperature) transformation; and (iii) the constant volume heat capacity exhibits finite jumps when entering and leaving the two-phase coexistence region.

These results have been applied to describe a prospective constant volume transition between liquid water and ice VI. By using thermodynamic information available from the literature, the transformation path in the p−T plane and the characteristic *F*, *S*, and $C_{V}$ curves as a function of temperature have been calculated and discussed. Additionally, and as a potential application, we have shown that a first-order phase transition under isochoric conditions could be used to drive a mechanical actuator controlled by temperature changes.

## Supplementary Materials

The following are available online at https://www.mdpi.com/article/10.3390/e24010031/s1, Figure S1: Gibbs energy change during a constant volume transformation between liquid water and ice VI, Figure S2: Internal energy change during a constant volume transformation between liquid water and ice VI, Figure S3: Enthalpy change during a constant volume transformation between liquid water and ice VI.

## Appendix A. Changes of G, U, and H during a Constant Volume Phase Transformation

In this Appendix, expressions for the changes of *G*, *U*, and *H* are calculated by using Equation (12), which is repeated here for the reader’s convenience:

(A1) $$\Delta Z\left(T\right)=Z\left(T\right)-Z_{m}^{\beta }\left(T_{1}\right)=Z_{m}^{\beta }\left(T\right)-Z_{m}^{\beta }\left(T_{1}\right)+\Delta_{PT}Z_{m}\left(T\right)\;\xi \left(T\right).$$

When applied to the Gibbs energy (Z=G), the contribution from the transformation is null (equality of chemical potentials), thus, the total change is given by:

(A2) $$\begin{array}{ccc} \Delta G(T) & = & G_{m}^{\beta }\left(T\right)-G_{m}^{\beta }\left(T_{1}\right)\\ & = & \left[F_{m}^{\beta }\left(T\right)+p_{c}\left(T\right)\;V_{m}^{\beta }\left(T\right)\right]-\left[F_{m}^{\beta }\left(T_{1}\right)+p_{c}\left(T_{1}\right)\;V_{m}^{\beta }\left(T_{1}\right)\right]\\ & = & F_{m}^{\beta }\left(T\right)-F_{m}^{\beta }\left(T_{1}\right)-{\left(\frac{\partial F_{m}^{\beta }}{\partial V_{m}}\right)}_{T}\left(T\right)\;V_{m}^{\beta }\left(T\right)+{\left(\frac{\partial F_{m}^{\beta }}{\partial V_{m}}\right)}_{T}\left(T_{1}\right)\;V_{m}^{\beta }\left(T_{1}\right). \end{array}$$

In the case of the internal energy *U*:

(A3) $$\begin{array}{ccc} \Delta U(T) & = & U_{m}^{\beta }\left(T\right)-U_{m}^{\beta }\left(T_{1}\right)+\Delta_{PT}U_{m}\left(T\right)\;\xi \left(T\right)\\ & = & \left[F_{m}^{\beta }\left(T\right)+TS_{m}^{\beta }\left(T\right)\right]\\ & & -\left[F_{m}^{\beta }\left(T_{1}\right)+T_{1}S_{m}^{\beta }\left(T_{1}\right)\right]+\left[\Delta_{t}F_{m}\left(T\right)+T\Delta_{PT}S_{m}\left(T\right)\right]\;\xi \left(T\right)\\ & = & F_{m}^{\beta }\left(T\right)-F_{m}^{\beta }\left(T_{1}\right)-T{\left(\frac{\partial F_{m}^{\beta }}{\partial T}\right)}_{V_{m}^{\beta }=V}\left(T\right)+T_{1}{\left(\frac{\partial F_{m}^{\beta }}{\partial T}\right)}_{V_{m}^{\beta }=V}\left(T_{1}\right)\\ & & +\left[\Delta_{t}F_{m}\left(T\right)+T\Delta_{PT}V_{m}\left(T\right)\frac{dp_{c}\left(T\right)}{dT}\right]\;\xi \left(T\right). \end{array}$$

For the enthalpy *H*:

(A4) $$\begin{array}{ccc} \Delta H(T) & = & H_{m}^{\beta }\left(T\right)-H_{m}^{\beta }\left(T_{1}\right)+\Delta_{PT}H_{m}\left(T\right)\;\xi \left(T\right)\\ & = & \left[U_{m}^{\beta }\left(T\right)+p_{c}\left(T\right)\;V_{m}^{\beta }\left(T\right)\right]-\left[U_{m}^{\beta }\left(T_{1}\right)+p_{c}\left(T_{1}\right)\;V_{m}^{\beta }\left(T_{1}\right)\right]\\ & & +\left[\Delta_{t}U_{m}\left(T\right)+p_{c}\left(T\right)\Delta_{PT}V_{m}\left(T\right)\right]\;\xi \left(T\right)\\ & = & F_{m}^{\beta }\left(T\right)-F_{m}^{\beta }\left(T_{1}\right)-T{\left(\frac{\partial F_{m}^{\beta }}{\partial T}\right)}_{V_{m}^{\beta }=V}\left(T\right)+T_{1}{\left(\frac{\partial F_{m}^{\beta }}{\partial T}\right)}_{V_{m}^{\beta }=V}\left(T_{1}\right)\\ & & +p_{c}\left(T\right)\;V_{m}^{\beta }\left(T\right)-p_{c}\left(T_{1}\right)\;V_{m}^{\beta }\left(T_{1}\right)+T\frac{dp_{c}\left(T\right)}{dT}\Delta_{PT}V_{m}\left(T\right)\;\xi \left(T\right). \end{array}$$

## Appendix B. Constant Volume Heat Capacity of the Whole System

Thermodynamic potentials are first-order homogeneous functions of their extensive variables. Hence, and according to Euler’s theorem, the total internal energy of a pure substance when phases β and γ coexist in equilibrium can be written as:

(A5) $$U\left(T,p,n^{\beta },n^{\gamma }\right)=U_{m}^{\beta }\left[p,T\right]\;n^{\beta }+U_{m}^{\gamma }\left[p,T\right]\;n^{\gamma },$$

where $U_{m}^{i}$ is the molar energy of phase *i* (i=β,γ). The constant volume specific heat of the whole system is:

(A6) $$\begin{array}{ccc} C_{V} & = & {\left(\frac{\partial U}{\partial T}\right)}_{V,n}\\ & = & n^{\beta }{\left(\frac{\partial U_{m}^{\beta }}{\partial T}\right)}_{V,n}+n^{\gamma }{\left(\frac{\partial U_{m}^{\gamma }}{\partial T}\right)}_{V,n}+U_{m}^{\beta }{\left(\frac{\partial n^{\beta }}{\partial T}\right)}_{V,n}+U_{m}^{\gamma }{\left(\frac{\partial n^{\gamma }}{\partial T}\right)}_{V,n}\\ & = & n^{\beta }{\left(\frac{\partial U_{m}^{\beta }}{\partial T}\right)}_{V,n}+n^{\gamma }{\left(\frac{\partial U_{m}^{\gamma }}{\partial T}\right)}_{V,n}+\left(U_{m}^{\beta }-U_{m}^{\gamma }\right){\left(\frac{\partial n^{\beta }}{\partial T}\right)}_{V,n}, \end{array}$$

since the total mole number $n=n^{\beta }+n^{\gamma }$ is fixed. The derivative of the single-phase molar energies reads:

(A7) $$\begin{array}{ccc} {\left(\frac{\partial U_{m}^{i}}{\partial T}\right)}_{V,n} & = & {\left(\frac{\partial U_{m}^{i}}{\partial T}\right)}_{p}+{\left(\frac{\partial U_{m}^{i}}{\partial p}\right)}_{T}{\left(\frac{\partial p}{\partial T}\right)}_{V,n}\\ & = & c_{p}^{i}-pV_{m}^{i}\alpha^{i}+V_{m}^{i}\left(\kappa^{i}p-\alpha^{i}T\right){\left(\frac{\partial p}{\partial T}\right)}_{V,n}, \end{array}$$

where $c_{p}^{i}$, $\alpha^{i}=\frac{1}{V_{m}^{i}}{\left(\frac{\partial V_{m}^{i}}{\partial T}\right)}_{p}$, and $\kappa^{i}=-\frac{1}{V_{m}^{i}}{\left(\frac{\partial V_{m}^{i}}{\partial p}\right)}_{T}$ are the constant pressure specific heat, the thermal-expansion coefficient, and the isothermal compressibility of the ith-phase, respectively. Additionally, ${\left(\frac{\partial p}{\partial T}\right)}_{V,n}=\frac{dp_{c}}{dT}$, as equilibrium requires. On the other hand, the conservation of the total volume is:

(A8) $$V_{0}=V\left(T,p,n^{\beta },n^{\gamma }\right)=V_{m}^{\beta }\left[p,T\right]\;n^{\beta }+V_{m}^{\gamma }\left[p,T\right]\;n^{\gamma }$$

resulting in:

(A9) $${\left(\frac{\partial n^{\beta }}{\partial T}\right)}_{V,n}=\frac{1}{V_{m}^{\beta }-V_{m}^{\gamma }}\left[-\left(n^{\beta }V_{m}^{\beta }\alpha^{\beta }+n^{\gamma }V_{m}^{\gamma }\alpha^{\gamma }\right)+\left(n^{\beta }V_{m}^{\beta }\kappa^{\beta }+n^{\gamma }V_{m}^{\gamma }\kappa^{\gamma }\right)\frac{dp_{c}}{dT}\right].$$

Inserting (A7) and (A9) in (A6) gives:

(A10) $$\begin{array}{cc} {\left(\frac{\partial U}{\partial T}\right)}_{V,n}= & \;n^{\beta }\left[c_{p}^{\beta }-pV_{m}^{\beta }\alpha^{\beta }+V_{m}^{\beta }\left(\kappa^{\beta }p-\alpha^{\beta }T\right)\frac{dp_{c}}{dT}\right]\\ \; & +\;n^{\beta }\left[c_{p}^{\gamma }-pV_{m}^{\gamma }\alpha^{\gamma }+V_{m}^{\gamma }\left(\kappa^{\gamma }p-\alpha^{\gamma }T\right)\frac{dp_{c}}{dT}\right]\\ \; & +\;\frac{\left(U_{m}^{\beta }-U_{m}^{\gamma }\right)}{V_{m}^{\beta }-V_{m}^{\gamma }}\left[-\left(n^{\beta }V_{m}^{\beta }\alpha^{\beta }+n^{\gamma }V_{m}^{\gamma }\alpha^{\gamma }\right)+\left(n^{\beta }V_{m}^{\beta }\kappa^{\beta }+n^{\gamma }V_{m}^{\gamma }\kappa^{\gamma }\right)\frac{dp_{c}}{dT}\right]. \end{array}$$

The molar energy difference can be written as:

(A11) $$U_{m}^{\beta }-U_{m}^{\gamma }=G_{m}^{\beta }-G_{m}^{\gamma }-p\left(V_{m}^{\beta }-V_{m}^{\gamma }\right)+T\left(S_{m}^{\beta }-S_{m}^{\gamma }\right).$$

Phase coexistence at equilibrium requires $G_{m}^{\beta }=G_{m}^{\gamma }$, and the Clapeyron equation gives $\frac{dp_{c}}{dT}=\frac{S_{m}^{\alpha }-S_{m}^{\beta }}{V_{m}^{\alpha }-V_{m}^{\beta }}$. Hence, Equation (A10) can be rewritten as:

(A12) $$\begin{array}{cc} {\left(\frac{\partial U}{\partial T}\right)}_{V,n}= & \;n^{\beta }\left[c_{p}^{\beta }-V_{m}^{\beta }\alpha^{\beta }T\;\frac{dp_{c}}{dT}\right]+n^{\gamma }\left[c_{p}^{\gamma }-V_{m}^{\gamma }\alpha^{\gamma }T\;\frac{dp_{c}}{dT}\right]\\ \; & +\;T\;\frac{dp_{c}}{dT}\left[-\left(n^{\beta }V_{m}^{\beta }\alpha^{\beta }+n^{\gamma }V_{m}^{\gamma }\alpha^{\gamma }\right)+\left(n^{\beta }V_{m}^{\beta }\kappa^{\beta }+n^{\gamma }V_{m}^{\gamma }\kappa^{\gamma }\right)\frac{dp_{c}}{dT}\right]. \end{array}$$

Since the constant pressure and constant volume specific heats are related by $c_{p}^{i}=c_{V_{m}^{i}}^{i}+\frac{TV_{m}^{i}{\alpha^{i}}^{2}}{\kappa^{i}}$, Equation (A12) becomes:

(A13) $$\begin{array}{cc} {\left(\frac{\partial U}{\partial T}\right)}_{V,n}= & \;n^{\beta }\left[c_{V_{m}^{\beta }}^{\beta }+\frac{TV_{m}^{\beta }\alpha {{}^{\beta }}^{2}}{\kappa^{\beta }}-2V_{m}^{\beta }\alpha^{\beta }T\;\frac{dp_{c}}{dT}+TV_{m}^{\beta }\kappa^{\beta }{\left(\frac{dp_{c}}{dT}\right)}^{2}\right]\\ \; & +\;n^{\gamma }\left[c_{V_{m}^{\gamma }}^{\gamma }+\frac{TV_{m}^{\gamma }\alpha {{}^{\gamma }}^{2}}{\kappa^{\gamma }}-2V_{m}^{\gamma }\alpha^{\gamma }T\;\frac{dp_{c}}{dT}+TV_{m}^{\gamma }\kappa^{\gamma }{\left(\frac{dp_{c}}{dT}\right)}^{2}\right], \end{array}$$

which finally can be conveniently rewritten to give:

(A14) $$\begin{array}{cc} {\left(\frac{\partial U}{\partial T}\right)}_{V,n}= & \;n^{\beta }\left[c_{V_{m}^{\beta }}^{\beta }+T\;V_{m}^{\beta }\;\kappa^{\beta }{\left(\frac{\alpha^{\beta }}{\kappa^{\beta }}-\frac{dp_{c}}{dT}\right)}^{2}\right]\\ \; & +\;n^{\gamma }\left[c_{V_{m}^{\gamma }}^{\gamma }+T\;V_{m}^{\gamma }\;\kappa^{\gamma }{\left(\frac{\alpha^{\gamma }}{\kappa^{\gamma }}-\frac{dp_{c}}{dT}\right)}^{2}\right]. \end{array}$$
