# Supplementary material for: First-Order Phase Transformation at Constant Volume: A Continuous Transition?
Source: Entropy (Basel). 2021 Dec 24;24(1):31. doi: 10.3390/e24010031 (PMC8774774; doi:10.3390/e24010031)
Supplement: Supplementary file 1 [file entropy-24-00031-s001.zip › entropy-1503426-supplementary.pdf]

Supplementary material. First-order transition at constant volume: a continuous transition?

V.F. Correa and F.J. Castro
Consejo Nacional de Investigaciones Científicas y Técnicas (CONICET)
Centro Atómico Bariloche (CNEA) and Instituto Balseiro (U. N. Cuyo)
Av. Bustillo 9500, 8400 Bariloche, Río Negro, Argentina

The first-order phase transition between liquid and ice-VI water phases is driven under icochoric condition ($V_0 = 13.4$ cm^3/mol). Figures S1, S2 and S3 show the corresponding changes in the Gibbs energy $\Delta G(T) = G(T) - G_m^{liq}(T_1)$, the internal energy $\Delta U(T) = U(T) - U_m^{liq}(T_1)$ and the enthalpy $\Delta H(T) = H(T) - H_m^{liq}(T_1)$, respectively. As predicted, all of them are continuous at T_1 and T_2, the temperature onset and end of the transition, respectively.

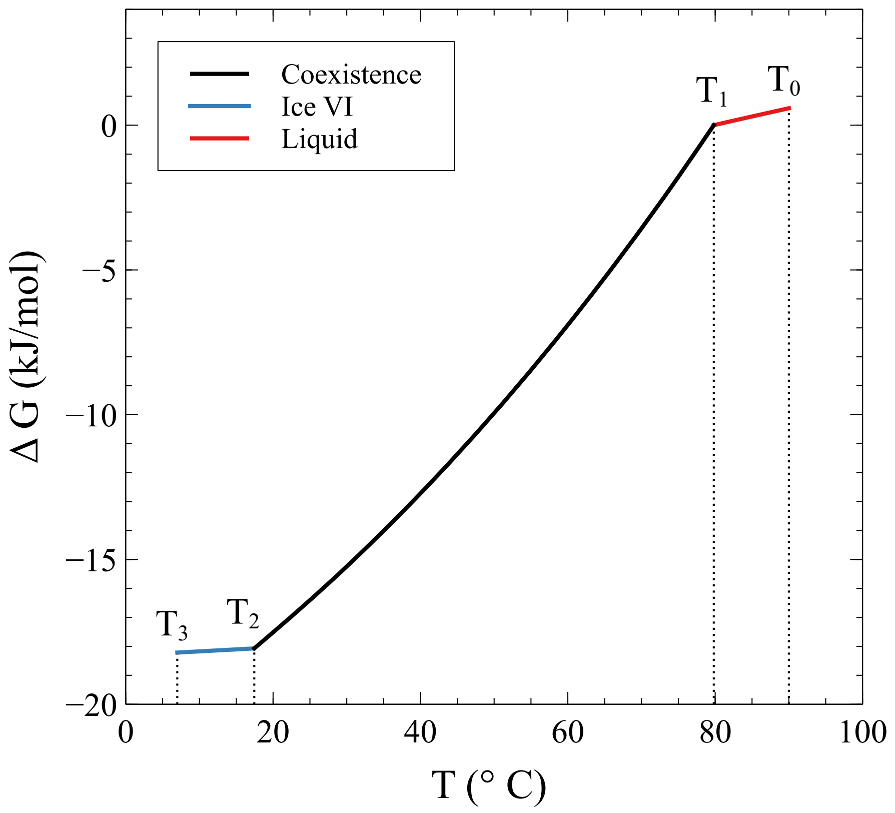

Figure S1: The Gibbs energy change $\Delta G(T) = G(T) - G_m^{liq}(T_1)$ in a process at constant volume $V_0 = 13.4\ \text{cm}^3/\text{mol}$.

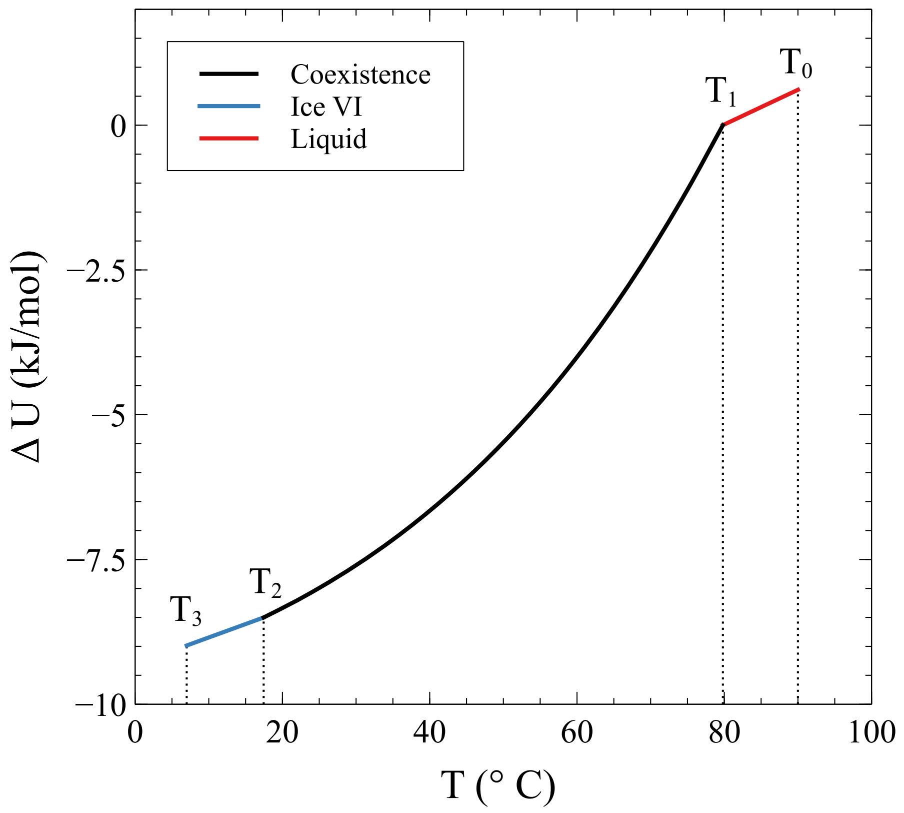

Figure S2: The internal energy change $\Delta U(T) = U(T) - U_m^{liq}(T_1)$ in a process at constant volume $V_0 = 13.4$ cm^3/mol.

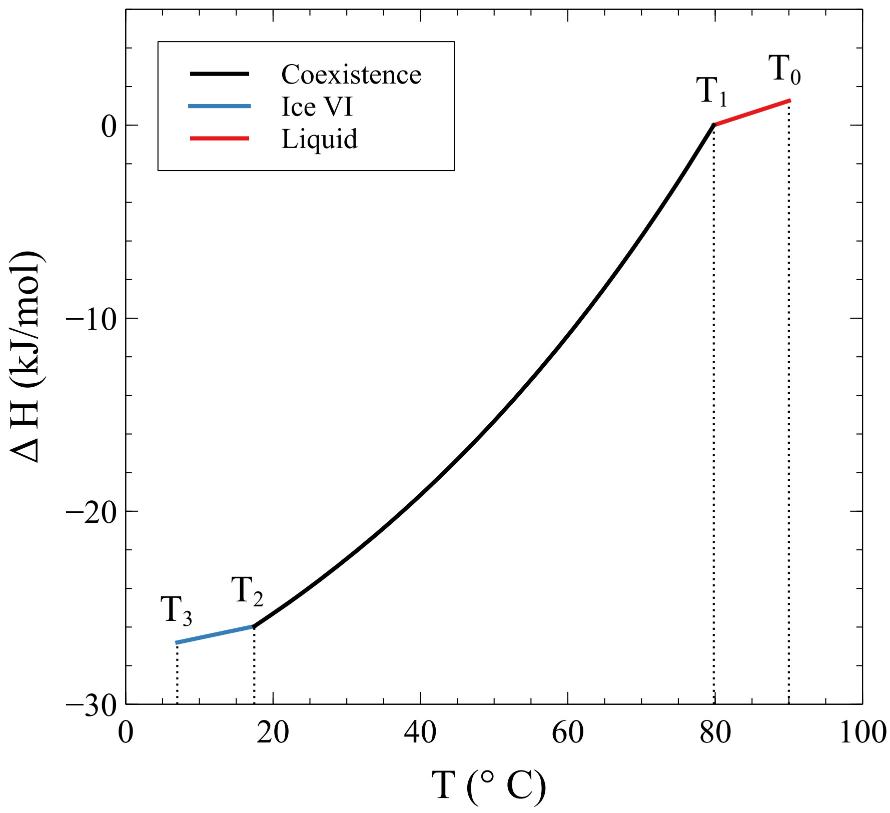

Figure S3: The enthalpy change $\Delta H(T) = H(T) - H_m^{liq}(T_1)$ in a process at constant volume $V_0 = 13.4$ cm^3/mol.
